# Abnormal Functional Connectivity of Resting State Network Detection Based on Linear ICA Analysis in Autism Spectrum Disorder

**DOI:** 10.3389/fphys.2018.00475

**Published:** 2018-05-08

**Authors:** Xia-an Bi, Junxia Zhao, Qian Xu, Qi Sun, Zhigang Wang

**Affiliations:** College of Mathematics and Computer Science, Hunan Normal University, Changsha, China

**Keywords:** linear independent component analysis, functional connectivity, autism spectrum disorder, neuro-pathophysiological mechanisms, resting state networks

## Abstract

Some functional magnetic resonance imaging (fMRI) researches in autism spectrum disorder (ASD) patients have shown that ASD patients have significant impairment in brain response. However, few researchers have studied the functional structure changes of the eight resting state networks (RSNs) in ASD patients. Therefore, research on statistical differences of RSNs between 42 healthy controls (HC) and 50 ASD patients has been studied using linear independent component analysis (ICA) in this paper. Our researches showed that there was abnormal functional connectivity (FC) of RSNs in ASD patients. The RSNs with the decreased FC and increased FC in ASD patients included default mode network (DMN), central executive network (CEN), core network (CN), visual network (VN), self-referential network (SRN) compared to HC. The RSNs with the increased FC in ASD patients included auditory network (AN), somato-motor network (SMN). The dorsal attention network (DAN) in ASD patients showed the decreased FC. Our findings indicate that the abnormal FC in RSNs extensively exists in ASD patients. Our results have important contribution for the study of neuro-pathophysiological mechanisms in ASD patients.

## Introduction

Autism spectrum disorder (ASD) is a developmental disorder. The symptoms of ASD are mainly manifested in social interaction and communication abnormalities, unusually repetitive patterns of behavior (Weng et al., 2010; von dem Hagen et al., 2013; Bos et al., 2014). ASD usually emerges in the early life, but sometimes accompanies the patient's life (Bookheimer et al., 2008; Allely, 2013). According to data released by the United States Centers for Disease Control in 2014, the probability of children being diagnosed with ASD is 1/68 in the United States, and the probability of a boy being diagnosed with ASD is 1/42 (Nevison, 2014; Alotaibi and Almalki, 2016). In contrast, the prevalence of autism children in the early 1970s was 1 in 2,500 (McDonald and Paul, 2010). The dramatic increase in ASD prevalence has led to an increasing number of scholars focusing on ASD (Hertzpicciotto and Delwiche, 2009; Neggers, 2014).

Although the etiology and pathogenesis of ASD are unclear (Careaga et al., 2013), the connectivity theory of ASD has been paid extensive attention in recent years (Geschwind and Levitt, 2007). Functional magnetic resonance imaging (fMRI) has played an important role in studying the abnormal neurobiological function in ASD (Iidaka, 2015). The advantage of fMRI is high time resolution and high spatial resolution (Goense et al., 2016). In addition, compared with other magnetic resonance imaging technology, such as positron emission tomography (PET) (Dichter, 2012), fMRI is non-invasive and does not rely on radiotracers. By employing fMRI into ASD, Rausch et al. (2016) studied the functional connectivity (FC) of amygdala in 20 ASD and 25 controls, and found that the FC was abnormal (Rausch et al., 2016). Olivito et al. (2017) found out the FC changes in the dentate nucleus and cerebral cortex in ASD patients (Olivito et al., 2017). Shen et al. (2016) concluded that the FC between amygdala and the brain regions responsible for social interaction had been destroyed in preschool-age children with ASD (Shen et al., 2016).

The above studies focus on the FC between the brain regions, but they do not provide the evaluation from the network connectivity level. Thus, Yerys et al. (2015) concluded that ASD patients displayed decreased and increased FC in default mode and non-default mode regions, respectively (Yerys et al., 2015). Padmanabhan et al. (2017) found the neurobiological features of ASD patients are the altered structure and function of default mode network (DMN), and the atypical developmental trajectory (Padmanabhan et al., 2017). Abbott et al. (2016) found that DMN and the right executive control network (ECN) in ASD patients were predominant over-connectivity, salience network and the left ECN in ASD patients were predominant under-connectivity (Abbott et al., 2016). In conclusion, the FC of these three networks in ASD patients is abnormal.

Many researchers focus on the FC of ASD patients in a single area, an interesting network or global connectivity features. But the eight resting state networks (RSNs) of ASD have not yet been studied. The neural mechanism of ASD could be better understood from the network connectivity and the resting state level. As one of the important network patterns, the eight RSNs have been used in social anxiety disorder (SAD) (Liao et al., 2010) and frontal lobe epilepsy (FLE) (Cao et al., 2014). Our paper focused on the eight RSNs of ASD and HC by employing the linear independent component analysis (ICA) which is introduced in the Appendix, and hypothesized that there were abnormalities in the FC of RSNs. We analyzed statistical differences of RSNs between 42 healthy controls (HC) and 50 ASD patients, and found that the RSNs with the decreased and increased FC in ASD patients included DMN, central executive network (CEN), core network (CN), visual network (VN), self-referential network (SRN). The RSNs with the increased FC in ASD patients included auditory network (AN), somato-motor network (SMN). The dorsal attention network (DAN) in ASD patients showed the decreased FC. The results of this study may provide significant contribution to study neuro-pathophysiological mechanisms in ASD patients.

## Materials and methods

### Subjects

The experimental data of this study were obtained from the open database Autism Brain Imaging Data Exchange (ABIDE) (http://fcon_1000.projects.nitrc.org/indi/abide/) (Di Martino et al., 2014). One hundred and seven subjects including 61 ASD patients and 46 HC were obtained. The ASD patients include 7 females and 54 males, whose ages are from 8 to 18 years old. The HC group includes 6 females and 40 males, whose ages are from 9 to 18 years old. The subject was excluded if the translation exceeded ±2.5 mm and rotation exceeded ±2.5. Finally, the remaining 92 subjects were involved in this study, including 50 ASD patients (age: 13.34 ± 2.41; 45 m/5 f) and 42 HC (age: 13.05 ± 1.82; 36 m/6 f).

**Figure 1:**
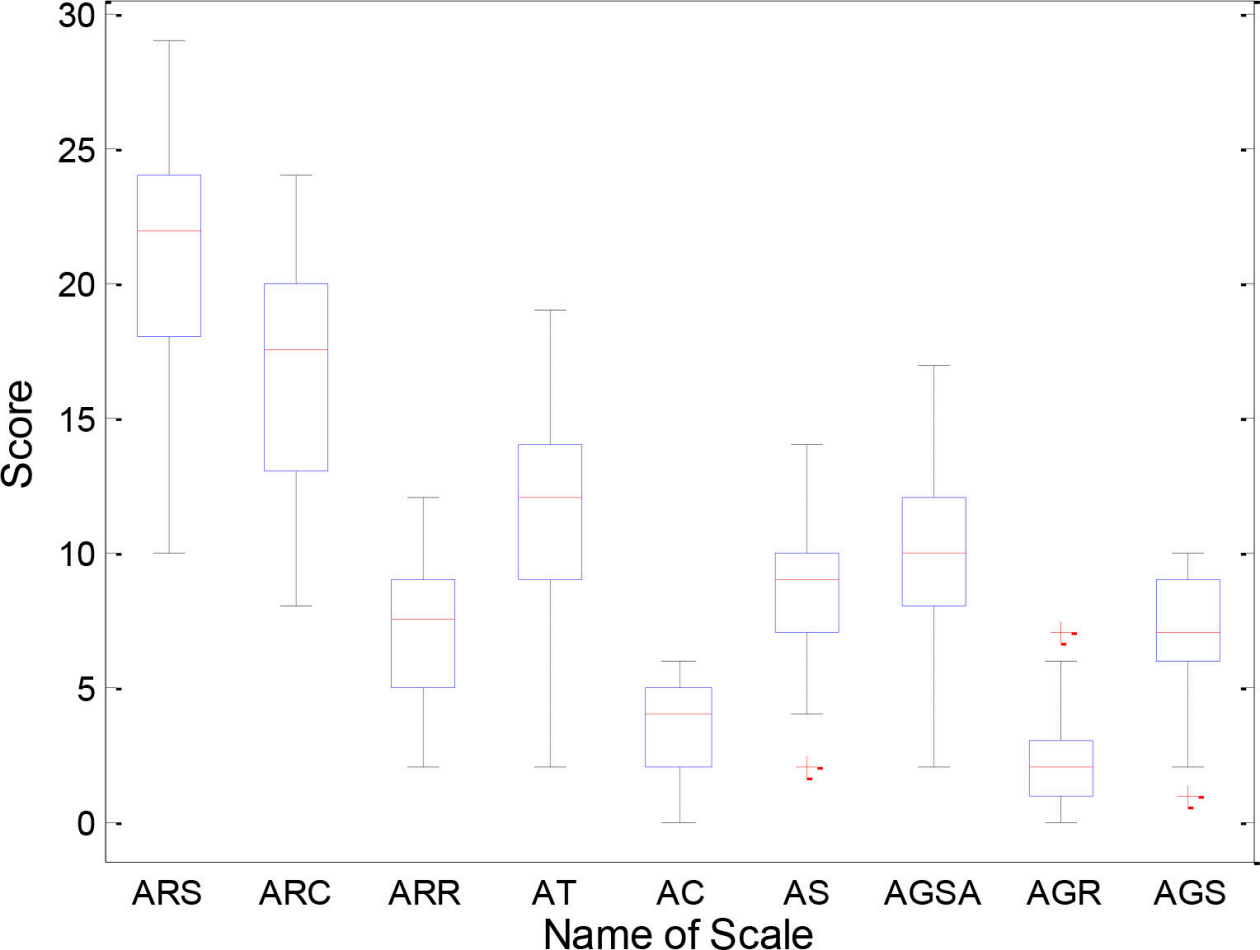
Distribution of scale values in ASD patients. ARS, ADI-R-Social; ARC, ADI-R-Communication; ARR, ADI-R-RRB; AT, ADOS-Total; AC, ADOS-Communication; AS, ADOS-Social; AGSA, ADOS-GOTHAM-Social-Affect; AGR, ADOS-GOTHAM-RRB; AGS, ADOS-GOTHAM-Severity.

We conducted chi-square test on the gender of the ASD patients and HC, and found no difference (*P* = 0.528). There was no difference (*P* = 0.520) in age between the two groups by two-sample *t-*tests. Clinical diagnosis of ASD was confirmed with the autism diagnostic interview-revised (ADI-R), the autism diagnostic observation schedule (ADOS) and the ADOS using Gotham algorithm. The demographic information for the ASD and HC groups is listed in Table 1 and Figure 1 shows the distribution of scale values in ASD patients.

**Table 1:** Demographic information of all subjects.

**Project**	**ASD (*n* = 50)**	**HC (*n* = 42)**	***P*-value**
Gender (Male/Female)	45/5	36/6	0.528^a^
Age	13.34 ± 2.41	13.05 ± 1.82	0.520^b^
Full IQ^c^	99.73 ± 14.40	107.21 ± 10.94	0.007^b^
ADI-R-Social	20.88 ± 4.68	**-**	**-**
ADI-R-Communication	16.88 ± 4.40	**-**	**-**
ADI-R-RRB	7.26 ± 2.41	**-**	**-**
ADOS-Total	11.48 ± 3.84	**-**	**-**
ADOS-Communication	3.42 ± 1.43	**-**	**-**
ADOS-Social	8.06 ± 2.71	**-**	**-**
ADOS-GOTHAM-Social-Affect^d^	9.43 ± 3.47	**-**	**-**
ADOS-GOTHAM-RRB^e^	2.43 ± 1.47	**-**	**-**
ADOS-GOTHAM-Severity^f^	6.83 ± 2.24	**-**	**-**

a The P-value is obtained through the chi-square test.

b *The P-value is obtained by the two-sample t-tests, and the data in the table is represented by the mean ± standard deviation*.

c *Score missing for one participant*.

d *Score missing for four participants*.

e *Score missing for four participants*.

f *Score missing for four participants*.

*ADR-R, autism diagnostic interview-revised; ADOS, autism diagnostic observation schedule; RRB, restricted and repetitive behaviors; ADOS-GOTHAM, standardized scores of ADOS using Gotham algorithm which has improved prediction capacity for ASD*.

### Image acquisition

Functional MRI data for all subjects were obtained from a 3.0T scanner of the Simens. The subjects were told to keep quiet, lie flat in the scanner, and try to stay still and not think about any problem. The sequence parameters corresponding to the functional images of all subjects were described as follows: TR = 3,000 ms, TE = 28 ms, flip angle = 90, matrix = 64 × 64, Pixel Spacing = 3.0 × 3.0, 0 mm thickness, without gap, number of volumes = 120, 34 slices.

### Data preprocessing

DPARSF software has helped us complete the data preprocessing (http://d.rnet.co/DPABI/DPABI_V2.3_170105.zip). The preprocessing platform is MATLAB (R2014a). Data preprocessing steps are as follows:

1. Format conversion: The original data collected from the database was in DICOM format which could not be recognized by the preprocessing software DPARSF. Thus, we converted the DICOM format of the original data to the NIFTI format.
2. Removing time points: As the scanner needs a certain amount of time to achieve a stable state, the first 10 volumes of fMRI data were discarded to make the scanner stable. Finally each subject had the 110 volumes.
3. Slice timing: As the data was not acquired from the same point in time, the remaining 110 volumes of each subject were corrected for the temporal difference in order to ensure that the data were collected at the same point in time.
4. Realigning: As the subjects have a slight translation or rotation in the scanning which produces image artifacts or errors, the head movement correction is needed to eliminate these errors. As a result, the head motion correction excluded 4 of the 46 healthy controls and 11 of the 61 ASD patients, because the translation exceeded ±2.5 mm and rotation exceeded ±2.5.
5. Normalization: Because the brain structure of each subject is different, the brains of each subject could not be compared. Therefore, the spatial normalization is conducted by using EPI templates to eliminate differences in individual brains.
6. Smoothing: Smoothing could reduce spatial noise and the difference between anatomical structures of the subjects. The data was smoothed by Gaussian kernel (*FWHM* = 6 mm).

### Determination of RSNs

This study used the linear ICA (Correa et al., 2007; Carnì et al., 2016) to extract the independent components (ICs) of ASD patients and HC by using GIFT software (http://icatb.sourceforge.net/, version 1.3e) (Calhoun et al., 2001; Liao et al., 2010). We first estimated the number of ICs of fMRI data for ASD patients and HC using the “Minimum description length (MDL)” criterion (Jafri et al., 2008). The MDL criterion is provided by the GIFT software. The process of extracting independent components is conducted by using Gift software. On the software interface, there is a question “Do you want to estimate the number of independent components,” and it should be chosen with “Yes.” Then the software interface will appear the sentence “the estimated independent components is found to be 27 using the MDL criteria” or “the estimated independent components is found to be 26 using the MDL criteria.” So, the number of ICs estimated in ASD patients was 26, and the number of ICs estimated in HC was 27.Secondly, principal component analysis was performed, which could reduce the temporal dimension of the fMRI data for ASD patients and HC. Finally, the ICs of ASD patients and HC were estimated by the fast ICA algorithm. The group spatial linear ICA was respectively carried out in the ASD group and the HC group. Then the 26 ICs in the ASD group and the 27 ICs in the HC group were obtained. These ICs include time-courses and spatial maps.

The time-courses and spatial maps of ICs reflect the waveform and intensity of brain activity, respectively (Mantini et al., 2007). It is generally believed that the *Z-*value is the most effective measure of the FC of the intrinsic network (Damoiseaux et al., 2006), thus we transformed the intensity value of the spatial map into the *Z-*value. By using this method, we could found out the voxels which have the greatest contribution to a specific IC (Calhoun et al., 2001). After obtaining the ICs in the two groups, we used the GIFT software to calculate the spatial correlation coefficients between the specific eight RSNs templates and ICs, and selected the IC of the largest spatial correlation coefficient (Greicius et al., 2007). The selected IC represents its corresponding RSN, and is retained for subsequent experiment. The eight RSN templates are provided by Dante Mantini from Leuven Medical School (Mantini et al., 2009) including DMN, DAN, AN, CN, SRN, SMN, VN, CEN.

### Two analysis methods for RSNs

After finding out the eight RSNs of ASD group and HC group using the largest spatial correlation principle (Greicius et al., 2007), the spatial maps corresponding to each RSNs of the two groups were collected to perform one-sample *t-*tests. The results of one-sample *t-*tests were presented at the given threshold of *T* > 2. Activation brain regions of RSNs could be obtained by one-sample *t-*tests. However, one-sample *t-*tests results only help to find out the activated brain regions, but could not be used to study the differences between the two groups. We further carried out two-sample *t*-tests, which could help us find the differences of RSNs between the two groups. The null hypothesis of the two-sample *t-*tests is that there are differences of the FC of RSNs in ASD group and HC group. Before the two-sample *t-*tests, we firstly clustered one-sample *t-*tests results into a union. Then the union was regarded as regions of interest (ROIs) which was utilized to calculate FC based on the voxels of 92 subjects, and the obtained FC was followed by the Z-transform. Finally, the two-sample *t-*tests were carried out, and the results were displayed at the given threshold of *P* < 0.05 (AlphaSim correction).

## Results

### Spatial pattern of RSNs in each group

**Figure 2:**
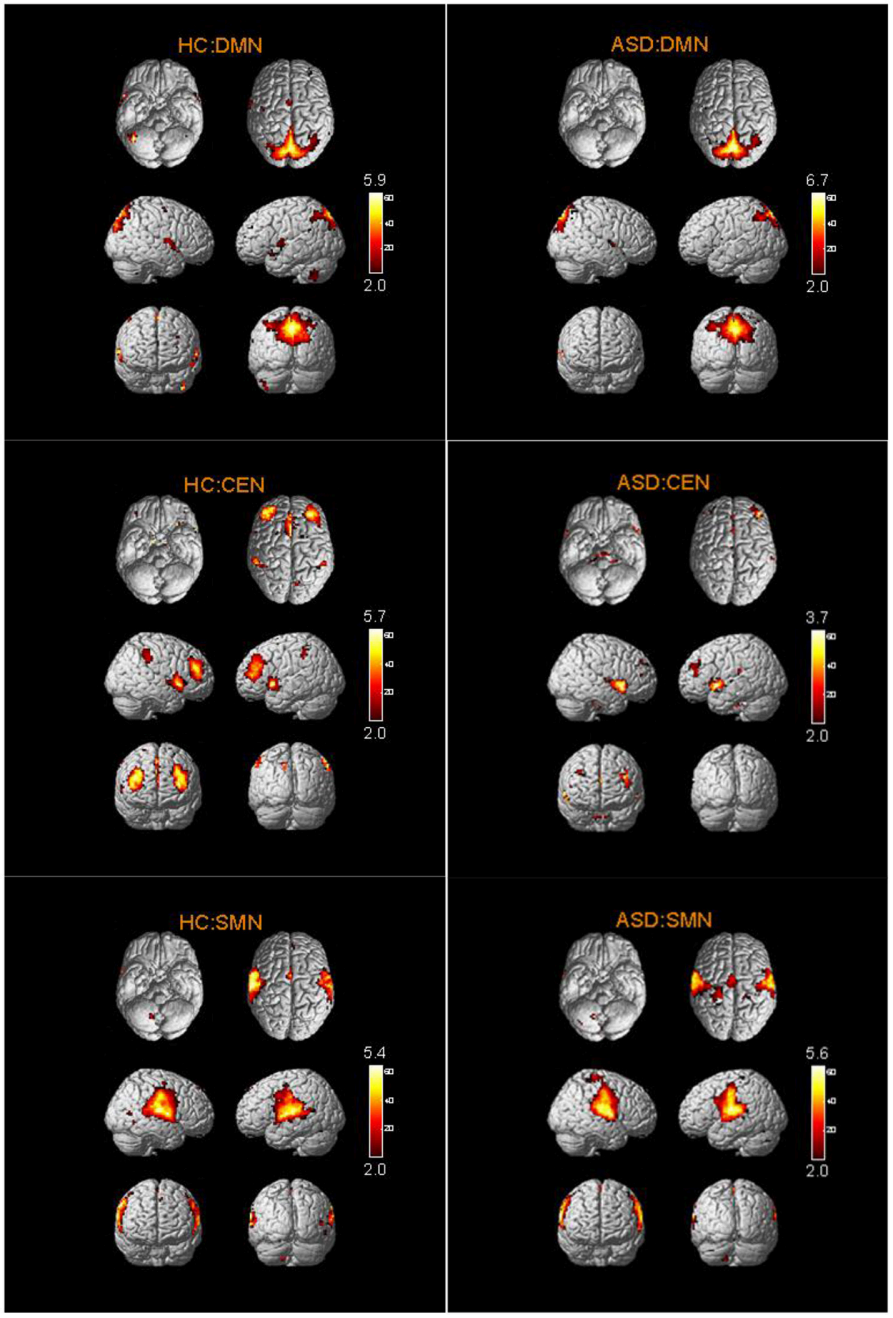
The spatial distribution of DMN, CEN, SMN in ASD group and HC group.

**Figure 3:**
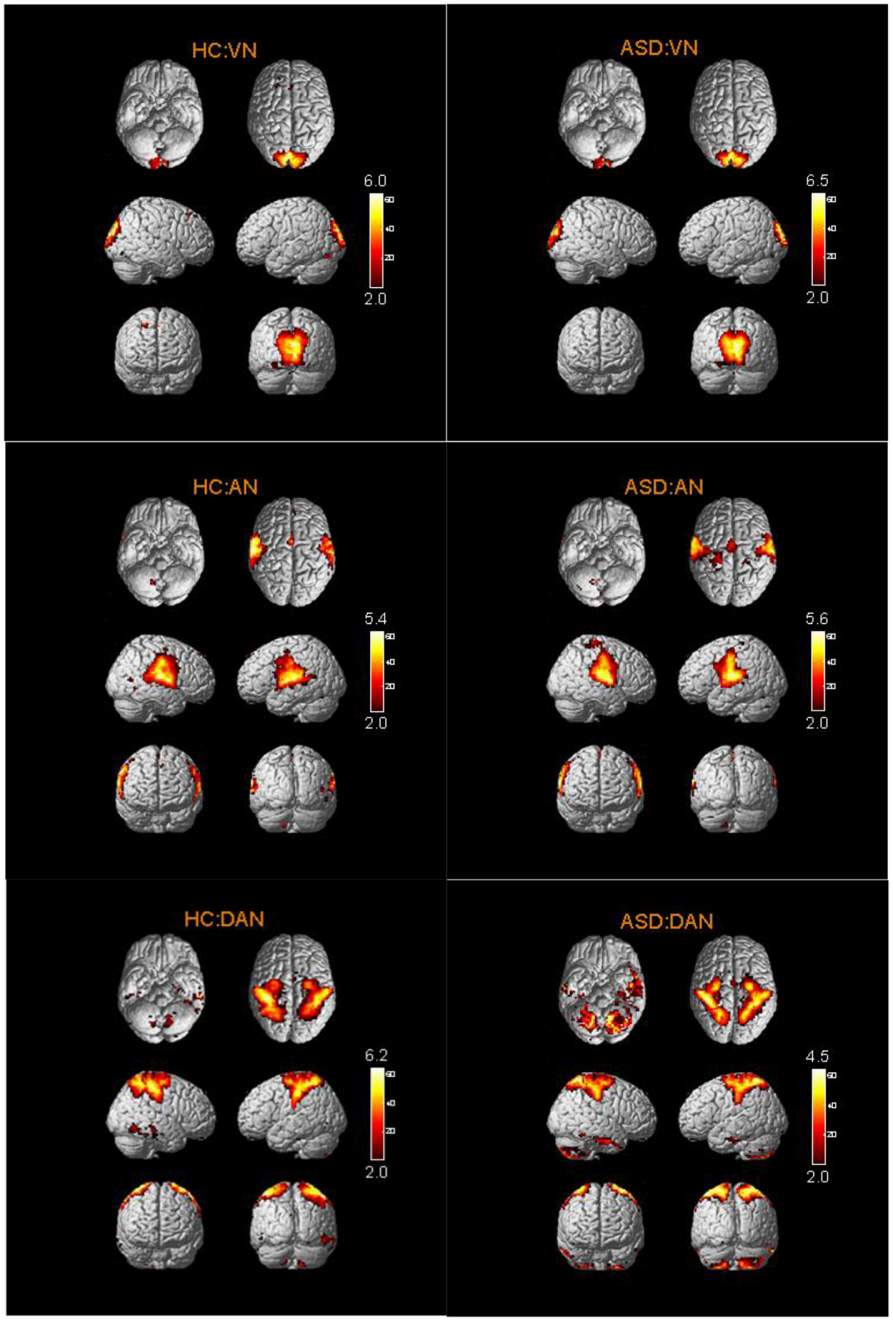
The spatial distribution of VN, AN, DAN in ASD group and HC group.

**Figure 4:**
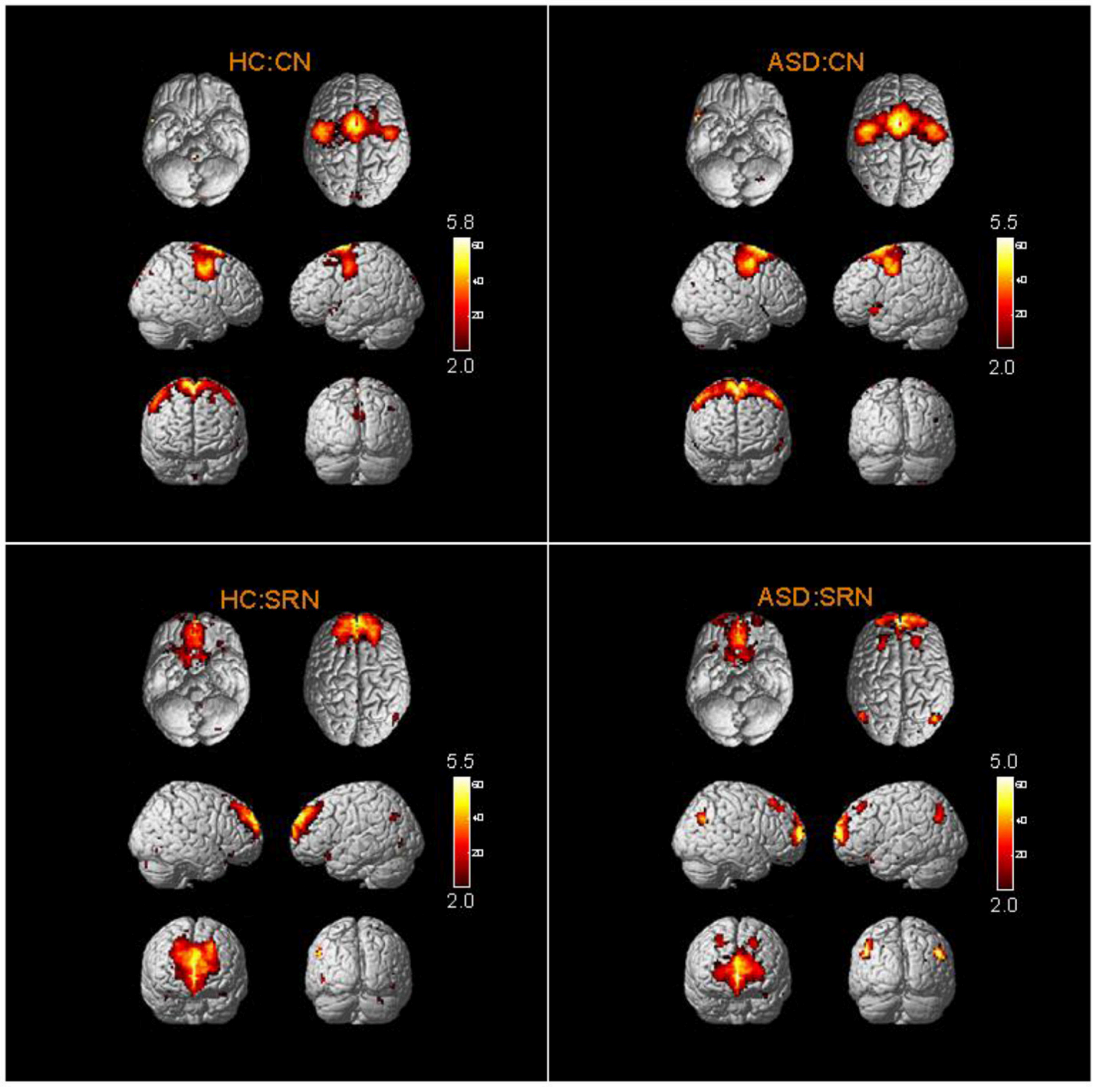
The spatial distribution of CN and SRN in ASD group and HC group.

The spatial distribution patterns of RSNs are shown by one-sample *t-*tests results (*T* > 2) in Figures 2–4. It can be seen from these figures that all subjects have a typical spatial distribution pattern of RSNs.

### Abnormal RSNs in ASD patients

**Figure 5:**
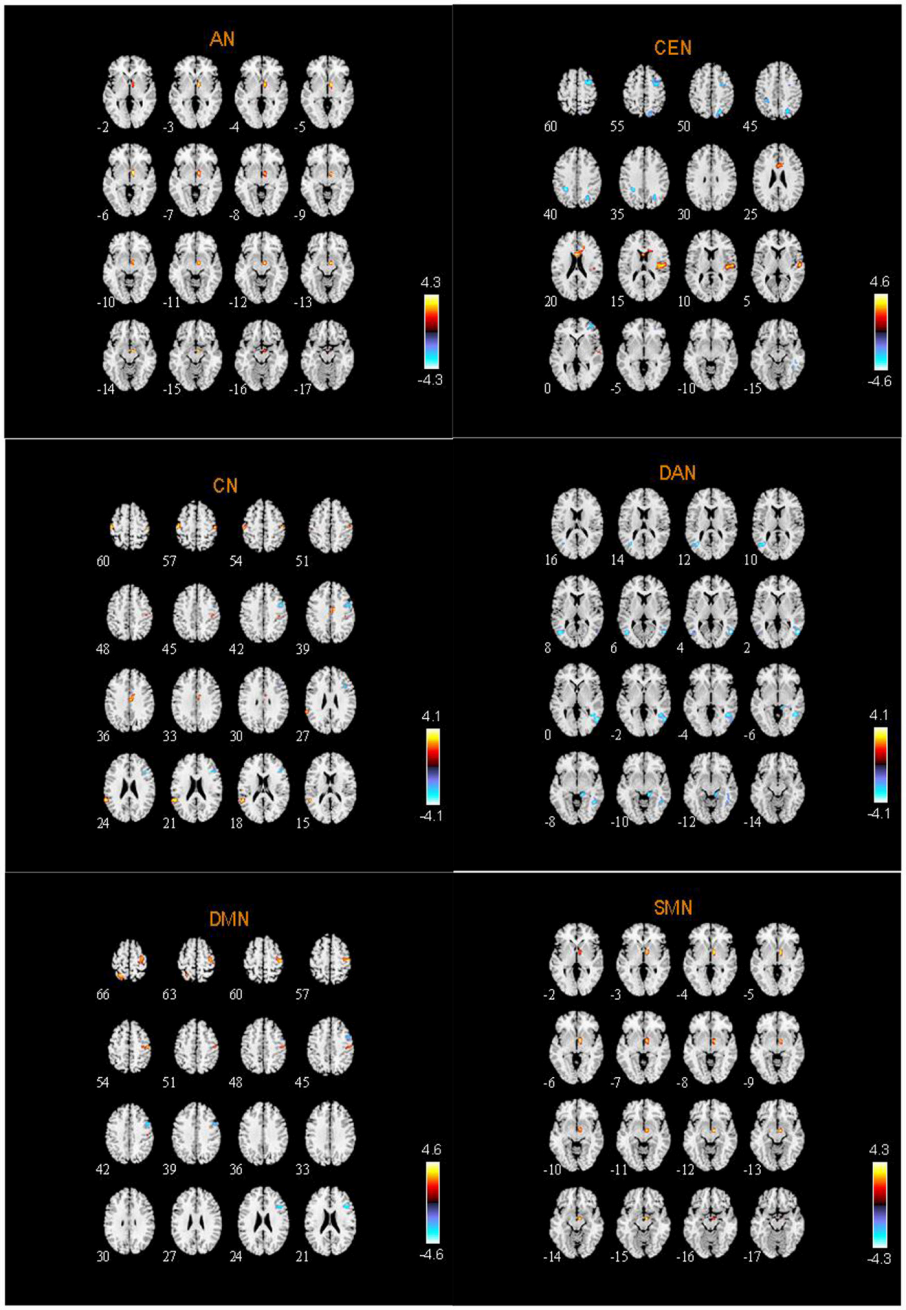
Abnormal brain regions in AN, CEN, CN, DAN, DMN, SMN.

**Figure 6:**
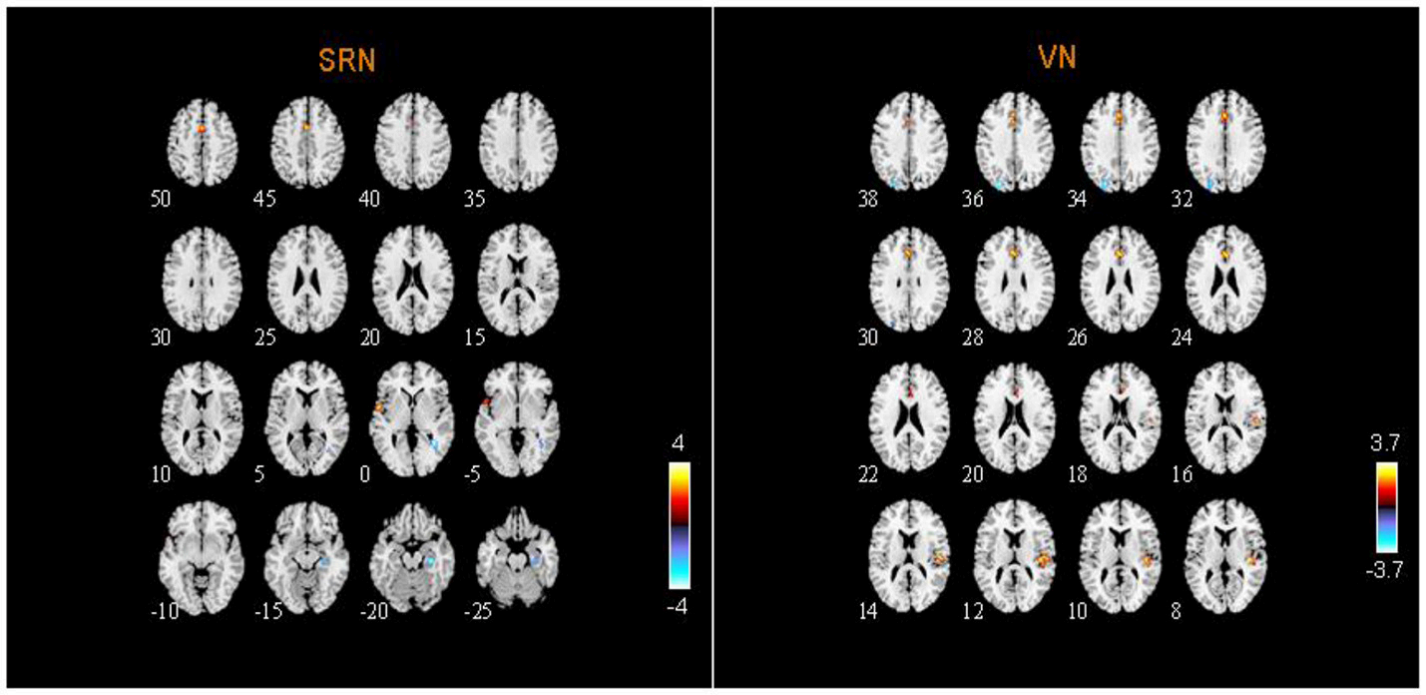
Abnormal brain regions in SRN and VN.

From two-sample *t-*tests results, we found out abnormal RSNs and brain regions between the ASD and HC groups as shown in Figures 5, 6. The RSNs with the decreased and increased FC (*P* < 0.05, AlphaSim corrected) in ASD patients included DMN, CEN, CN, VN, SRN. The RSNs with the increased FC (*P* < 0.05, AlphaSim corrected) in ASD patients included AN, SMN. The DAN in ASD patients showed the decreased FC (*P* < 0.05, AlphaSim corrected).

Specifically, Table 2 shows the clusters with significant differences of the FC in RSNs of ASD patients. Compared to HC, the increased and decreased FC in ASD patients are all the abnormal FC. The abnormal brain regions of the FC in DMN are situated in the right hemisphere including triangular part of inferior frontal gyrus (IFGtriang), middle frontal gyrus (MFG), precentral gyrus (PreCG), postcentral gyrus (PoCG), and left hemisphere including superior parietal gyrus (SPG) and precuneus (PCUN).

**Table 2:** Clusters with significant differences of functional connectivity in RSNs of ASD.

**Cluster**	**Abnormal the brain regions**	**The number of voxel**	**Peak coordinates**
**DMN**			
Cluster1	IFGtriang.R	47	[51 21 21]
Cluster2	PreCG.R MFG.R	39	[54 9 42]
Cluster3	PoCG.R	105	[48 −21 57]
Cluster4	SPG.LPCUN.L	49	[−18 −60 69]
**AN**			
Cluster1	PAL.R	46	[9 0 −6]
**CEN**			
Cluster1	FFG.RITG.R	37	[42 −36 −21]
Cluster2	STG.RINS.R	118	[60 −12 3]
Cluster3	ACG.RACG.L	94	[0 12 18]
Cluster4	SPG.RSOG.RMOG.R	122	[24 −72 51]
Cluster5	MFG.R SFGdor.R	125	[33 3 57]
**CN**			
Cluster1	STG.L	54	[−63 −45 24]
Cluster2	IFGtriang.R	38	[42 24 21]
Cluster3	DCG.R	32	[48 3 42]
Cluster4	PreCG.R	32	[3 −75 33]
Cluster5	PoCG.R	33	[51 −21 57]
Cluster6	PoCG.L PreCG.L	62	[−45 −27 66]
**DAN**			
Cluster1	MTG.R ITG.R	129	[57 −66 3]
Cluster2	PHG.R LING.R	30	[15 −36 −9]
Cluster3	MTG.L	58	[−36 −66 12]
**SMN**			
Cluster1	PAL.R	46	[9 0 −6]
**SRN**			
Cluster1	PHG.R	47	[33 −24 −21]
Cluster2	STG.L	34	[−54 15 −6]
Cluster3	SMA.L SMA.R	72	[3 9 48]
**VN**			
Cluster1	IOG.R FFG.R	48	[30 −84 −15]
Cluster2	STG.R HES.R	47	[45 −21 9]
Cluster3	ACG.L DCG.L ACG.R	64	[−3 30 27]
Cluster4	SOG.L MOG.L	39	[−24 −81 36]

The abnormal brain regions of the FC in CEN are situated in the right hemisphere including fusiform gyrus (FFG), inferior temporal gyrus (ITG), MFG, middle occipital gyrus (MOG), dorsolateral of superior frontal gyrus (SFGdor), superior temporal gyrus (STG), insula (INS), and the bilateral anterior cingulate and paracingulate gyri (ACG).

The abnormal brain regions of the FC in CN are situated in the right hemisphere including IFGtriang, median cingulate and paracingulate gyri (DCG), and left hemisphere including STG, and the bilateral PreCG and PoCG.

The abnormal brain regions of the FC in VN are situated in the right hemisphere including inferior occipital gyrus (IOG), FFG, STG, ACG, and left hemisphere including superior occipital gyrus (SOG), MOG, ACG, DCG.

The abnormal brain regions of the FC in DAN are situated in the right hemisphere including ITG, parahippocampal gyrus (PHG), lingual gyrus (LING), and the bilateral middle temporal gyrus (MTG).

SRN includes the right PHG, the left STG and the bilateral supplementary motor area (SMA). AN and SMN include the right pallidum (PAL).

## Discussion

In this paper, we analyzed the fMRI data of ASD and HC groups using linear ICA. We studied the difference of the eight RSNs between ASD group and HC group. Our findings concluded that the RSNs with the decreased and increased FC in ASD patients include DMN, CEN, CN, VN, SRN, and the increased FC includes AN, SMN, and the decreased FC includes DAN.

CEN is responsible for human cognitive control related to emotional or non-emotional materials (Miller and Cohen, 2001; Ochsner and Gross, 2005). The abnormal brain regions of the FC in CEN include INS, ACG, MFG, and SFGdor. Firstly, the frontal lobe locates in the front of the human brain. Its executive function includes cognitive activity, emotional activity, the ability to predict future results from current behavior (Alvarez and Emory, 2006; Lau, 2013; Watanabe et al., 2015). Meanwhile, it is related to human long-term memory in resting state (Neulinger et al., 2016). Hitherto, there have been several studies focusing on SFGdor of ASD (Assaf et al., 2010; Rudie et al., 2012). It is suggested that the FC of SFGdor decreases in ASD patients compared with HC (Assaf et al., 2010). We also found that the FC of both MFG and SFGdor decreased in ASD patients, which is associated with social and communication deficits in ASD patients (Assaf et al., 2010). Secondly, the cingulate gyrus is mainly concerned with self-instruction, self-awareness, and self-control (Redcay, 2008). It is showed that the dysfunction of ACG in ASD patients is related to the symptoms of behavioral disorders (Thakkar et al., 2008). Our results also indicated that the FC of ACG was abnormal, which is related to behavioral disorders in ASD patients (Hoffmann et al., 2016). Thirdly, INS is the source of social emotion (Pavuluri and May, 2015). It is found that INS activation and connectivity were aberrant in ASD (Odriozola et al., 2016). In this paper, it was also discovered that the FC of INS was abnormal.

The brain region with the abnormal FC in AN and SMN is PAL. PAL is a part of the basal ganglia (Groenewegen, 2003; Nelson and Kreitzer, 2014). Lesions of the basal ganglia are able to cause a variety of motor and cognitive disorders (DeLong and Georgopoulos, 2011; Bekiesinska-Figatowska et al., 2013). Furthermore, the structures of subcortical regions in ASD patients were also studied, and it is found that the shape of PAL changes with age in ASD, which is closely related to the abnormal behavior (Schuetze et al., 2016). Our findings also showed that the FC of PAL was abnormal, which is consistent with existing studies (Schuetze et al., 2016; Turner et al., 2016).

DMN is closely related to human advanced cognitive activities, including internal psychological activities, environmental monitoring, and episodic memory retrieval (Vincent et al., 2006). The abnormal brain regions of the FC in DMN are mainly the right PreCG, PoCG, MFG and the left SPG, PCUN. Specifically, PCUN, PoCG, and SPG belong to the parietal lobe whose main role is to integrate sensory information, such as processing tactile and visual space information (Gentile et al., 2011). We found out the abnormalities of the FC in PCUN, PoCG, and SPG caused by impaired information integration in ASD patients. Other abnormal brain regions of the FC in DMN include MFG and PreCG. Firstly, MFG is mainly responsible for the coordination of different information (Japee et al., 2015). Previous studies have suggested that ASD patients are not complete in information processing and cannot integrate and process information, thereby they cannot communicate normally in public places (Skoyles, 2011; Mohd Roffeei et al., 2015). Our results revealed the decreased FC in MFG, which is related to the communication disorders in ASD patients. On the other hand, one of the components of the primary motor cortex is PreCG (Yeo et al., 2014). Our study found out the abnormal FC in PreCG, which has made a significant contribution to the relationship between ASD's athletic and social abilities (Nebel et al., 2014).

The abnormal brain regions of the FC in CN are mainly located in STG, PoCG, PreCG. STG is not only involved in auditory processing but also involved in social cognition (Bigler et al., 2007). The activation of STG was significantly reduced in ASD patients, which was discovered by the researchers (Kana et al., 2016). Our results also showed the abnormal FC of the left STG in ASD patients. This finding suggests a possible failure of language function in left hemisphere, which is related to STG in ASD patients.

DAN is concerned with the regulation of goal-directed top-down processing (Corbetta and Shulman, 2002). According to our results, the abnormal brain regions of the FC in DAN include MTG, ITG, and LING. The temporal lobe is divided into STG, MTG, and ITG, and it is involved in social cognition. Specifically, it is responsible for processing auditory information and also related to memory and emotion (Amlerova et al., 2014). It is concluded that the structure of MTG is abnormal in ASD patients (Salmond et al., 2005). Our study also found that the FC of both ITG and MTG decreased in ASD patients, which provides a functional explanation for the temporal lobe abnormalities in ASD patients. The other abnormal brain region of the FC in DAN is LING. LING is mainly responsible for visual processing (Yang et al., 2015). We found that the FC of LING decreased in ASD patients, and we infer that this may be related to social interaction impairment of ASD patients.

The abnormal brain regions of the FC in SRN include PHG, STG, and SMA. It is showed that the abnormal FC of PHG in ASD patients is related to the restriction and repetitive behavior (Monk et al., 2009). Our findings also suggested that the FC of PHG in ASD patients was abnormal, which is consistent with previous studies. The relationship between STG and the symptoms of ASD patient has been described in the above. However, the relationship between SMA and the symptoms of ASD patients is not clear and needs further study.

The abnormal brain regions of the FC in VN include STG, ACG, DCG, IOG, MOG, and SOG. The occipital lobe is the center of visual information processing (Wandell et al., 2007; Johnson et al., 2015). When the occipital lobe is damaged, it causes not only visual impairment, but also memory impairment and motion perception disorders (Larsson and Heeger, 2006; Scahill et al., 2013). Previous studies have concluded that the visual perception construction dysfunction in ASD patients may be a clinical manifestation of “occipital-temporal” dysfunction (Griffiths and Milne, 2007; Baum et al., 2015). Our results also indicate that the FC of STG, IOG, MOG, and SOG were abnormal. Thus, our findings provide an explanation for the visual impairment in ASD patients from the neurological function view.

In conclusion, there are two main reasons for the abnormal FC in ASD patients. On the one hand, it is associated with the clinical symptoms of ASD patients. The main clinical symptoms of ASD include impairment in social interaction and difficulties in communication, unusually repetitive patterns of behavior. For example, the brain regions that play an important role in social communication and interaction include MTG, FFG, amygdala, medial prefrontal cortex, inferior frontal gyrus (IFG). The abnormal FC of these regions is related to the impairment in social interaction and difficulties in communication of ASD patients (Philip et al., 2012; Kim et al., 2015). The abnormal FC of IFG, STG is related to the defects in social language processing and social attention of ASD patients (Redcay, 2008). The frontal lobe, STG, parietal cortex, and amygdala might mediate impairments of social behaviors (Adolphs, 2001; Kim et al., 2010) and the orbitofrontal cortex (OFC) and caudate nucleus have been associated with restricted and repetitive behaviors of ASD (Atmaca et al., 2007).

On the other hand, it is related to the brain structure of ASD patients. Such as the FFG structure of ASD is asymmetrical (Dougherty et al., 2016); Gray and white matter are abnormal in ASD (Sungji et al., 2015); There are differences in the total brain volume between ASD and HC (Lange et al., 2015). Brain volume abnormalities include increased volume of frontal and temporal lobes in early brain development in ASD patients (Nordahl et al., 2011) and the brain volume increases with age in younger ASD patients (Courchesne et al., 2011).

## Ethics statement

This study was carried out in accordance with the recommendations of Health Insurance Portability and Accountability Act (HIPAA) guidelines, National Institutes of Health (NIH) Combined Neuroscience Institutional Review Board with written informed consent from all subjects. All subjects gave written informed consent in accordance with the Declaration of Helsinki. The protocol was approved by the National Institutes of Health (NIH) Combined Neuroscience Institutional Review Board.

## Author contributions

XB: proposed the design of the work and revised it critically for important intellectual content; QS and QX: carried out the experiment for the work and drafted part of the work; JZ and ZW: collected, interpreted the data, and drafted part of the work. All the authors approved the final version to be published and agreed to be accountable for all aspects of the work in ensuring that questions related to the accuracy or integrity of any part of the work are appropriately investigated and resolved.

### Conflict of interest statement

The authors declare that the research was conducted in the absence of any commercial or financial relationships that could be construed as a potential conflict of interest.
