# Supplementary material for: Abnormal Functional Connectivity of Resting State Network Detection Based on Linear ICA Analysis in Autism Spectrum Disorder
Source: Front Physiol. 2018 May 8;9:475. doi: 10.3389/fphys.2018.00475 (PMC5952255; doi:10.3389/fphys.2018.00475)
Supplement: Supplementary file 1 [file Data_Sheet_1.docx]

Appendix

**Linear ICA and its application in fMRI**

**Principles of linear ICA method**

The mathematical model of the linear ICA method is

$X=AS$ (1)

$X={（x_{1} ,x_{2} ,\ldots,x_{n}）}^{T}$ is the observation signal, and its dimension is N. A is the mixed matrix of $M\times N$. $S={（s_{1} ,s_{2} ,\ldots s_{m}）}^{T}$is the unknown source signal of M-dimensional. The matrix expression of Equation 4 is as follows:

$\left[ \begin{aligned} x_{1} \\ \vdots\\ x_{n} \end{aligned} \right]=\left[ \begin{matrix} a_{11} & \cdots& a_{1m} \\ \vdots& \vdots& \vdots\\ a_{n1} & \cdots& a_{\mathrm{nm}} \end{matrix} \right]\left[ \begin{aligned} s_{1} \\ \vdots\\ s_{m} \end{aligned} \right]$ (2)

In summary, the principle of ICA is to estimate the unknown mixed matrix $A$ and *S* under the condition that the observed signal is known.

Figure 1 shows the ICA model.

***Y*=B*X***

***X*=A*S***

***S***

B

A

Figure 1 The ICA model

Some assumptions and constraints on the ICA model are as follows:

1. It is assumed that the independent component (IC) is statistically independent.

2. IC must obey non-Gaussian distribution.

3. It is assumed that the mixed matrix is square matrix.

When the above three constraints are met, a standard ICA is obtained. Then it is able to calculate the unmixing matrix B, which is the purpose of ICA. According to the unmixing matrix B, we could calculate $Y={（y_{1} ,y_{2} ,\ldots,y_{n}）}^{T}$, which is the estimate of *S*. The formula is as follows:

$Y=BX=\tilde{S}$ (3)

where *Y*is IC, that is, an estimate of *S*.

**Applications of linear ICA in fMRI**

The application of ICA in fMRI mainly includes time ICA (tICA) and spatial ICA

(sICA). The application of tICA and sICA in fMRI are as follows:

1. The application of sICA in fMRI: The sICA regards image data obtained at each time point as a mixed component. When the scan time is N, there are N mixed components. After performing sICA, a series of independent images and corresponding time series can be obtained. The image corresponds to the activation of the brain region, while the time series corresponds to the intensity of the neural activity that the brain region.

It is assumed that fMRI image sequences include $M$images, and each image has $n$ voxels, and the i-th image is denoted by $m_{i}$. The process of sICA is as follows:

$\left[ \begin{aligned} m_{1} \\ \vdots\\ m_{N} \end{aligned} \right]$=$\left[ \begin{matrix} x_{11} & \ldots& x_{1m} \\ \vdots& \vdots& \vdots\\ x_{N1} & \cdots& x_{\mathrm{Nm}} \end{matrix} \right]$ (4)

$\left[ \begin{aligned} m_{1} \\ \vdots\\ m_{N} \end{aligned} \right]=M_{s}\left[ \begin{aligned} C_{s1} \\ \vdots\\ C_{sL} \end{aligned} \right]$ (5)

where$m_{i}$ indicates the intensity of $m$voxels at a certain point in time. $\left[ \begin{matrix} C_{s1} & \cdots& C_{sL} \end{matrix} \right]^{T}$ is spatial IC.

2. The application of tICA in fMRI: Time ICA regards time series obtained at N time points as a mixed component. After performing tICA, a series of independent images and corresponding time series can be obtained.

It is assumed that fMRI image sequences include $M$images, and each image has $n$ voxels, the time series of voxel i is $t_{i}$. The process of tICA is as follows:

$\left[ \begin{aligned} t_{1} \\ \vdots\\ t_{N} \end{aligned} \right]$=$\left[ \begin{matrix} x_{11} & \ldots& x_{1m} \\ \vdots& \vdots& \vdots\\ x_{N1} & \cdots& x_{Nm} \end{matrix} \right]$ (6)

$\left[ \begin{aligned} t_{1} \\ \vdots\\ t_{N} \end{aligned} \right]=M_{t}\left[ \begin{aligned} C_{t1} \\ \vdots\\ C_{tL} \end{aligned} \right]$ (7)

$\left[ \begin{matrix} t_{1} & \cdots& t_{N} \end{matrix} \right]^{T}$ represents the time course of a voxel. Each row of the$\left[ \begin{matrix} x_{11} & \ldots& x_{1m} \\ \vdots& \vdots& \vdots\\ x_{N1} & \cdots& x_{Nm} \end{matrix} \right]$ represents the time course.$\left[ \begin{aligned} C_{t1} \\ \vdots\\ C_{tL} \end{aligned} \right]$ represents the time IC. The schematic diagram of these two methods is shown in Figure 2.

Data Matrix

Time process

Voxel number

=

Mixed matrix

*

components

A voxel's time course

Time series

Spatial IC

sICA

Data Matrix

Time course

Voxel number

=

Mixed matrix

*

components

A voxel's time course

spatial pattern

Time IC

tICA

Figure 2 The sICA and tICA
